# TDP-43, an ALS Linked Protein, Regulates Fat Deposition and Glucose Homeostasis

**DOI:** 10.1371/journal.pone.0071793

**Published:** 2013-08-13

**Authors:** Nancy R. Stallings, Krishna Puttaparthi, Katherine J. Dowling, Christina M. Luther, Dennis K. Burns, Kathryn Davis, Jeffrey L. Elliott

**Affiliations:** 1 Department of Neurology and Neurotherapeutics, The University of Texas Southwestern Medical Center, Dallas, Texas, United States of America; 2 Department of Pathology, the University of Texas Southwestern Medical Center, Dallas, Texas, United States of America; 3 Department of Plastic Surgery, the University of Texas Southwestern Medical Center, Dallas, Texas, United States of America; Universitat de Barcelona, Spain

## Abstract

The identification of proteins which determine fat and lean body mass composition is critical to better understanding and treating human obesity. TDP-43 is a well-conserved RNA-binding protein known to regulate alternative splicing and recently implicated in the pathogenesis of amyotrophic lateral sclerosis (ALS). While TDP-43 knockout mice show early embryonic lethality, post-natal conditional knockout mice show weight loss, fat depletion, and rapid death, suggesting an important role for TDP-43 in regulating energy metabolism. Here we report, that over-expression of TDP-43 in transgenic mice can result in a phenotype characterized by increased fat deposition and adipocyte hypertrophy. In addition, TDP-43 over-expression in skeletal muscle results in increased steady state levels of Tbc1d1, a RAB-GTPase activating protein involved in Glucose 4 transporter (Glut4) translocation. Skeletal muscle fibers isolated from TDP-43 transgenic mice show altered Glut4 translocation in response to insulin and impaired insulin mediated glucose uptake. These results indicate that levels of TDP-43 regulate body fat composition and glucose homeostasis in vivo.

## Introduction

Tat activating regulatory DNA-binding protein (TDP-43) is a ubiquitously expressed and well conserved DNA/RNA binding protein that is believed to regulate alternative splicing and microRNA biogenesis [1–4]. TDP-43 contains two RNA binding domains that preferentially bind UG repeats of large target transcripts which in mammals include the cystic fibrosis transmembrane conductance regulator (CFTR) and survival for motor neuron (SMN). Steady state levels of TDP-43 are tightly regulated within cells via an auto feedback loop in which TDP-43 actively binds its own mRNA, and enhances the splicing of an intron in the 3’ untranslated region leading to nonsense mediated RNA degradation [5,6]. TDP-43 normally localizes to the nucleus, but in sporadic amyotrophic lateral sclerosis (ALS), both full length TDP-43 and small molecular weight TDP-43 immuno-reactive species aberrantly localize to extra-nuclear regions [7,8]. Mutations in the TDP-43 gene cause an autosomal dominant familial form of amyotrophic lateral sclerosis (ALS10) clearly establishing a link between TDP-43 and the human disease [9–12]. Overexpression of either wild type (WT) or mutant human TDP-43 within the CNS leads to a progressive motor phenotype which shares similar pathologic features with human disease, while high expression of TDP-43 in skeletal muscle produces a severe myopathy characterized by the accumulation of TDP-43 positive inclusions [13–15].

In contrast to over-expression, the conventional knockout of TDP-43 in mice is early embryonic lethal [16–18]. Therefore, to gain insight into possible actions of TDP-43 in vivo, Chiang et al., generated an inducible conditional TDP-43 knockout mouse model in which TDP-43 could be targeted in older mice [19]. The global post-natal deletion of TDP-43 resulted in rapid loss of body fat, weight loss and lethality. Screening studies showed that the loss of TDP-43 resulted in depressed levels of mRNA and protein for Tbc1d1, a key regulator of glucose 4 transporter (Glut4) translocation in skeletal muscle that is linked to human familial obesity [20,21]. Further experiments to confirm the physiological impact of lower TDP-43 levels in skeletal muscle were not reported, but these results suggested that TDP-43 may be a powerful regulator of lean/fat body mass in vivo.

In order to further test this hypothesis, we assessed the effects of TDP-43 over-expression on body fat and glucose metabolism in vivo. Here we show using specific lines of transgenic mice that TDP-43 over-expression leads to early onset enhanced fat mass and marked abnormalities in skeletal muscle glucose uptake in response to insulin. These results validate the hypothesis that TDP-43 is a key regulator of body fat composition and glucose homeostasis in vivo.

## Results

### A315T TDP-43 mice have increased fat mass

**Figure 1:**
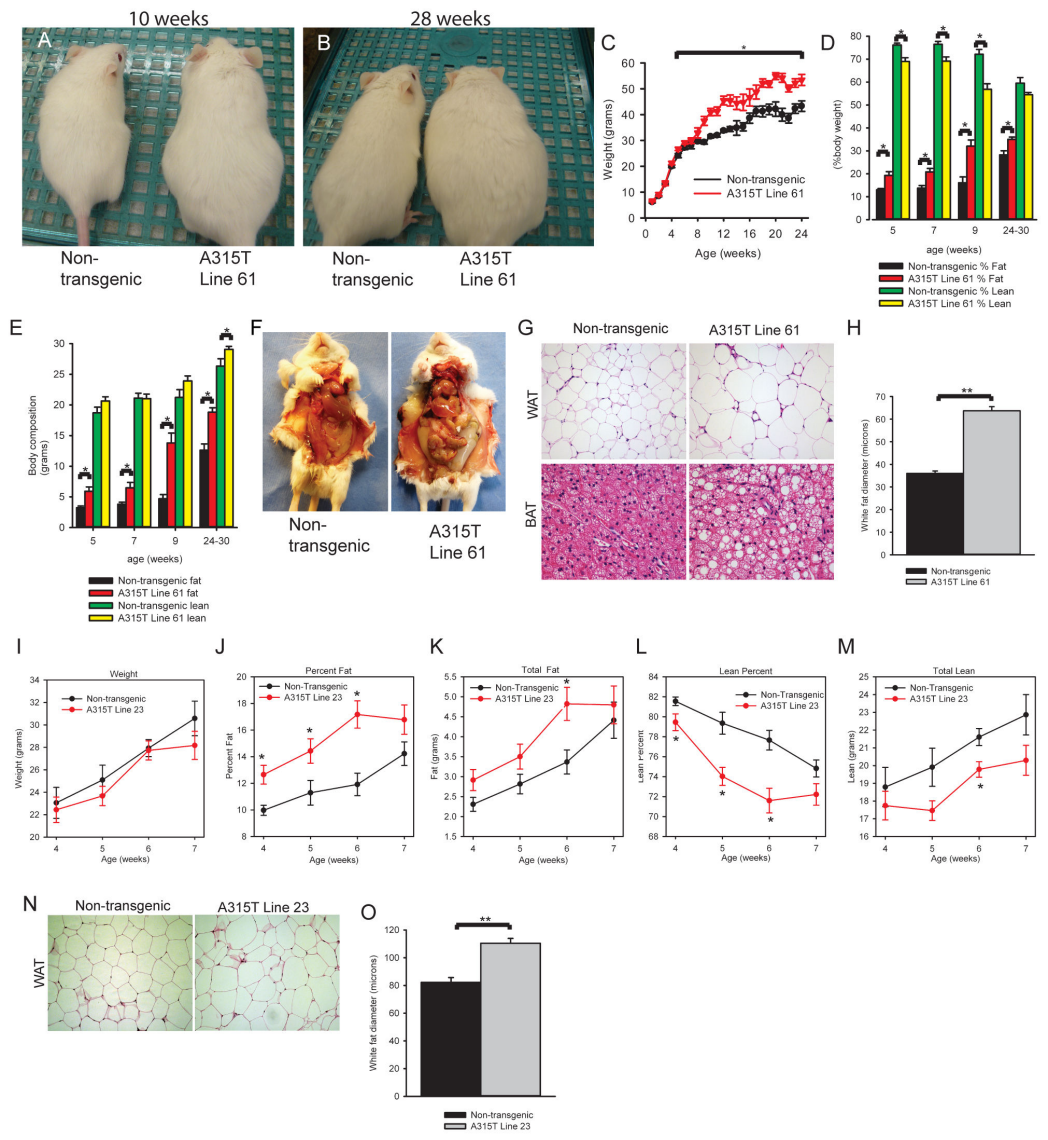
A315T TDP-43 mice develop marked obesity with a concomitant increase in adipose and decrease in lean mass percentage. (**A** and **B**) Increased size in A315T line 61 mice compared to non-transgenic littermates at 10 weeks (**A**) and 28 weeks (**B**). (**C**) Weight curves of A315T line 61 and non-transgenic mice (N ≥ 4). (**D**–**E**) Body composition analysis as measured by Echo MRI at 5, 7, 9, and 24-30 weeks for A315T line 61 and non-transgenic mice (N ≥3). Percent fat and lean mass (**D**). Total grams fat and lean mass (**E**). (**F**) Fat pad deposition at 16 weeks in non-transgenic and A315T line 61. (**G**) H&E of non-transgenic and A315T line 61 mice (16 weeks) in both WAT and BAT. Magnification: 200X (WAT) and 400X (BAT). (**H**) Quantification of white adipocyte size in non-transgenic and A315T line 61 (16 weeks). N=58-63 cells. (**I**) Weekly weights of TDP-43 A315T Line 23 and non-transgenic mice. (**J**–**M**) Body composition analysis as measured by Echo MRI for A315T Line 23 and non-transgenic mice, percent fat (**J**), total fat (**K**), percent lean body composition (**L**), and total lean grams (**M**). N=10-14 mice per group. (**N**) H&E of non-transgenic and A315T line 23 mice (6 weeks of age) in WAT. Magnification: 200X. (**O**) Quantification of white adipocyte size in non-transgenic and A315T line 23 (6 weeks). N=66-70 cells. Data are means ±S.E.M. Statistical significance between non-transgenic and A315T mice or between indicated conditions: *P<0.05. **P<.001.

While monitoring lines of TDP-43 transgenic mice for neurological disease and weakness, we observed that line 61 mice, expressing a human familial ALS linked A315T mutation, became much larger than non-transgenic litter mates and appeared to develop progressive obesity without manifesting neurological deficits (Figure 1, A and B). Body weight curves for line 61 transgenics and non-transgenic controls were similar until 6 weeks of age but then diverged, with transgenics gaining significantly more weight (Figure 1C). Body composition analysis by MRI spectroscopy revealed a significant increase in the percentage of body fat with a corresponding decrease in percentage of lean body mass by 5 weeks of age (Figure 1D). Total body fat is also significantly increased in line 61 mice by five weeks compared to non-transgenics while total lean mass is unchanged from controls at these early time points (Figure 1E). These data demonstrate that the increase in weight of line 61 mice derives from increased fat mass. Necroscopy confirmed an increase in abdominal fat pad deposition in line 61 transgenic mice compared to controls (Figure 1F). Histological examination of white adipose tissue (WAT) and brown adipose tissue (BAT) showed marked increase in fat storage for transgenics (Figure 1G). Adipocytes in white fat were significantly larger in A315T line 61 transgenics than in non-transgenics (Figure 1H). Because of the obvious increase in fat deposition and adipocyte hypertrophy, we asked whether adipose tissue in line 61 mice showed abnormal remodeling and expansion. However, a panel of chemokine, cytokine, pro-inflammatory and metalloproteinase markers associated with abnormal adipocyte expansion was not significantly different than non-transgenics as assessed by quantitative real time PCR (Figure S1) [22]. These results indicate that line 61 TDP-43 transgenic mice develop marked progressive weight gain, increased body fat, and adipocyte hypertrophy.

We then asked whether another line of A315T TDP-43 transgenic mice showed similar changes in body fat composition. A315T TDP-43 line 23 mice develop an early onset severe motor neuron disease (ALS) phenotype that has been previously characterized [13]. We only assessed early time points (4-7 weeks old) in these mice as the development of paralysis eventually limits caloric intake and survival. In contrast to line 61 mice, line 23 mice do not have increased weight compared to non-transgenic controls (Figure 1I). However, MRI spectroscopy performed in A315T TDP-43 line 23 mice before onset of motor deficits, showed significantly increased percentage of body fat, total body fat, decreased lean mass percentage, and total lean mas compared to age and sex matched non-transgenic controls as early as 4 weeks of age (Figure 1J–M). Histological examination of white adipose tissue (WAT) from A315T line 23 showed marked increase in fat storage for transgenics (Figure 1N). Adipocytes in white fat were significantly larger in A315T line 23 transgenics than in non-transgenics (Figure 1O). Results from these two lines of mice suggest that the increased fat deposition in TDP-43 transgenic mice appears independent of the motor neuron disease phenotype or weight differential from control. Thus over-expression of human A315T TDP-43 in mice leads to significant changes in body fat composition.

### Adipose and muscle in A315T line 23 and line 61 have increased TDP-43 levels

**Figure 2:**
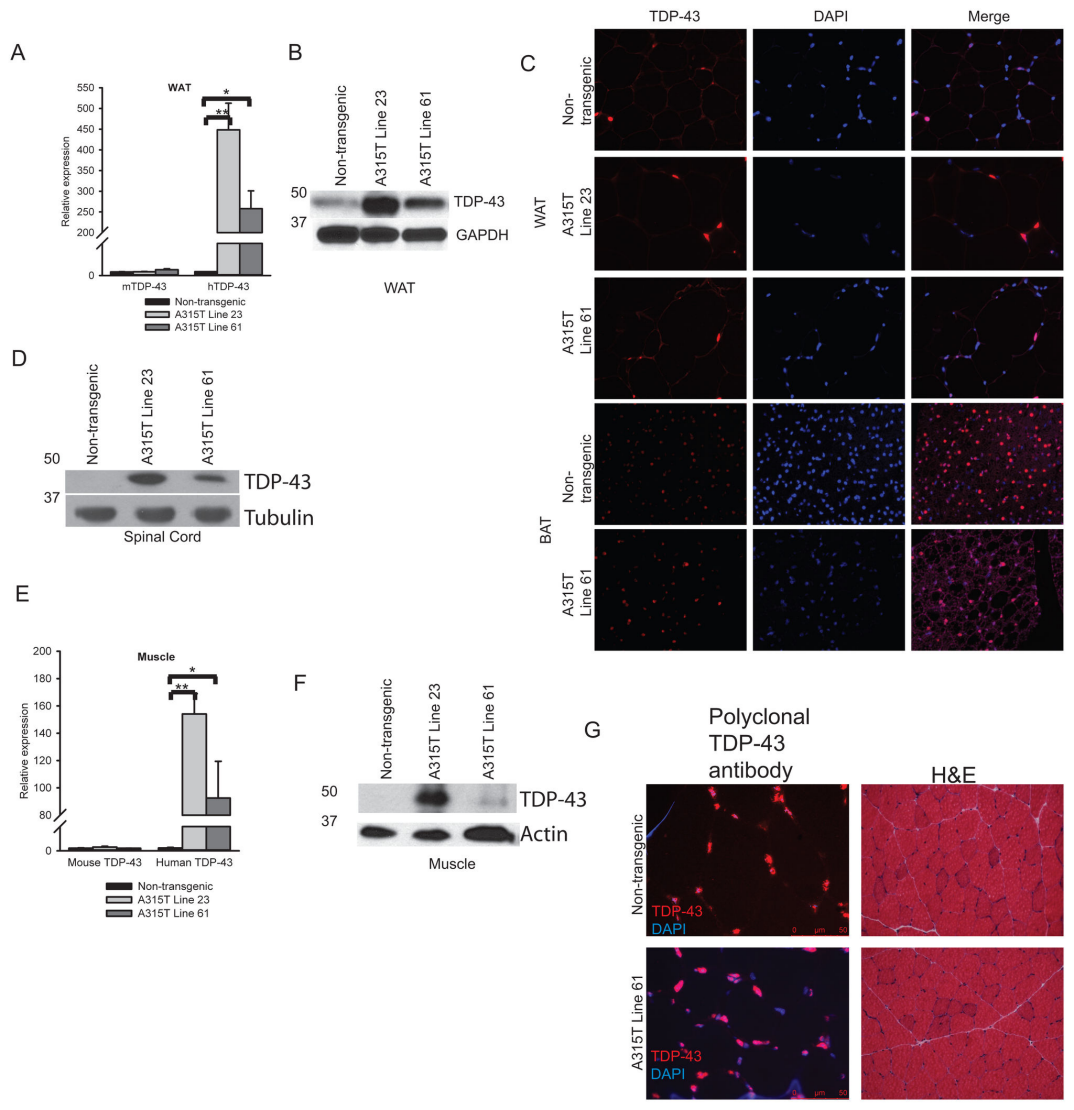
White adipose tissue and muscle in A315T line 23 and line 61 have increased TDP-43 levels. (**A**) Quantification of TDP-43 endogenous and transgene mRNA levels in WAT (6 weeks, N=4). Data are fold gene expression normalized with cyclophilin and expressed as mean ± S.E.M. *P<0.05, **P<0.001. (**B**) TDP-43 protein levels in WAT from 6 week old mice probed with polyclonal antibody that recognizes both human and mouse TDP-43. (**C**) TDP-43 immuno-reactivity in WAT and BAT of non-transgenic (16 weeks). A315T line 23 (6 weeks) and A315T line 61 mice (16 weeks). Magnification=400X. (**D**) TDP-43 protein levels in spinal cord from non-transgenic, A315T line 23 and A315T line 61(12 week old) mice using monoclonal antibody which recognizes human TDP-43 only. (**E**) Quantification of TDP-43 endogenous and transgene mRNA levels in skeletal muscle (6 weeks, N=4). Data are fold gene expression normalized with cyclophilin and expressed as mean ± S.E.M. *P<0.05, **P<0.005. (**F**) TDP-43 protein levels in skeletal muscle of non-transgenic, A315T line 23, A315T line 61 mice (12 weeks of age) using polyclonal antibody. (**G**) TDP-43 immunoflorescence and H&E stained sections of skeletal muscle of non-transgenic and A315T line 61 (16 weeks of age). Magnification: 400X for immunoflorescence,200X for H&E. 5 µg of total protein loaded per lane for spinal cord and muscle western blot. 10 µg of total protein loaded per lane for WAT western blot. 1 µg of total protein loaded per lane for muscle western blot. Tubulin, actin or GAPDH were used as loading controls. Representative western blots are shown, each sample was run ≥ 2 times with N≥ 2 mice in each group analyzed.

We then sought to better characterize the patterns of TDP-43 transgene expression in line 23 and line 61 in various tissues. In WAT from abdominal fat pads, TDP-43 mRNA and protein steady state levels were increased in both line 23 and line 61 mice compared to non-transgenic controls, but TDP-43 retained its normal nuclear localization (Figure 2, A-C). Expression levels of both mRNA and protein of TDP-43 were greater in line 23 compared to line 61. Western blots showed significantly less total TDP-43 in spinal cords extracts from line 61 compared to A315T TDP-43 line 23 which develops marked neurogenic weakness and spinal cord pathology as previously published (Figure 2D) [13]. In concordance with low line 61 spinal cord expression of TDP-43 and normal motor phenotype, histological examination of A315T line 61 spinal cords appeared normal, without evidence of ubiquitin inclusions, neuronal loss or gliosis, similar to non-transgenics (Figure S2). TDP-43 mRNA levels in skeletal muscle were elevated in line 23 and line 61 mice compared to non-transgenics (Figure 2E). In skeletal muscle TDP-43 protein levels were lower in line 61 than in the ALS model line 23 (Figure 2F) [13]. On immuno-staining, TDP-43 localization appeared strictly nuclear in line 61 muscle (Figure 2G). In agreement, histological examination of skeletal muscle from line 61 obese mice revealed normal muscle, indistinguishable from that of non-transgenics (Figure 2G). Skeletal muscle pathology in line 23, which develops weakness, shows denervation and atrophy as previously published [13]. Thus, levels of TDP-43 expression in line 23 and line 61 mice are elevated in white adipose tissue, spinal cord, and skeletal muscle compared to non-transgenic mice. In all tissues examined, line 23 mice had higher levels of TDP-43 than line 61. This data suggests that spinal cord expression levels of line 61 are below thresholds needed to generate pathology and motor phenotypes. Both line 23 and line 61 show similar adipose pathology suggesting that the phenotype seen is not due to an aberrant transgene insertion site in either line.

### Metabolic parameters in A315T Line 61 transgenic and non-transgenic mice

**Figure 3:**
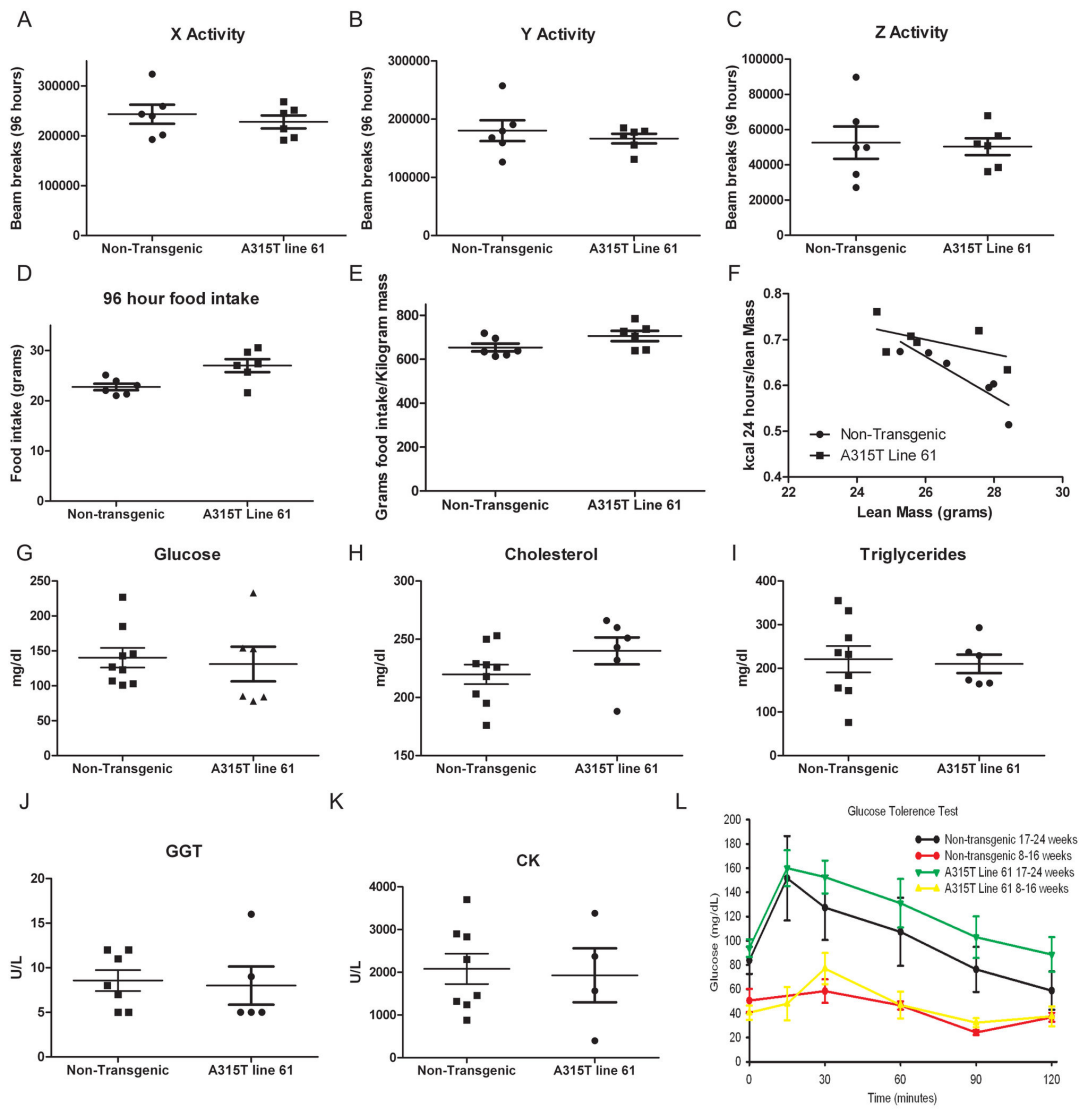
Metabolic analysis of A315T line 61 mice. (**A**–**D**) Cumulative values from individual mice over 96 hours for horizontal X/Y activity (**A** and **B**), vertical Z activity (**C**) and food intake (**D**) from non-transgenic and A315T line 61 mice in metabolic cages. (**E**) Food intake normalized to weight for A315T line 61 and non-transgenic controls for a 96 hour period. (**F**) Energy expenditure normalized to lean mass (10 weeks of age; N=6 each group). Bar =mean±S.E.M. (**G**-**K**) Fasting blood values for glucose (**G**), cholesterol (**H**), triglycerides (**I**), gamma glutamyltransferase (**J**), and creatine kinase (**K**) in non-transgenic and A315T line 61 mice (8-16 weeks, N= 4-9). (**L**) Glucose tolerance test in non-transgenic and A315T line 61 mice (N=5-12).

We performed further studies on line 61 mice to evaluate whether they manifested alterations in metabolic parameters. For line 61 mice, overall spontaneous locomotor activity as measured by beam breaks in the X, Y, and Z axis was unchanged from non-transgenic mice (Figure 3, A-C). Although cumulative food intake for line 61 mice was increased when compared to controls for the 96 hour period measured, when corrected for weight there was no significant difference (Figure 3 D-E). Energy expenditure when corrected for lean mass was not significantly different between A315T line 61 and non-transgenic mice (Figure 3F, Figure S3A, Table S1). Of note, although the transgenic and non-transgenic mice used in the metabolic cages had different weights, the amount of lean mass was unchanged between the groups (Figure S3 B–D). Blood chemistries showed no differences in glucose, cholesterol, triglycerides, gamma glutamyltransferase (GGT) and creatine kinase (CK) levels between fasting line 61 mice and controls (Figure 3, G-K). Glucose tolerance testing (GTT) in young (<16 weeks of age) was similar in both 61 transgenics and controls (Figure 3L). Older transgenic and control mice (>17 weeks) showed an initial similar peak glucose level 15 minutes after challenge. Although not reaching significance, older transgenic mice trended toward higher blood glucose values at 30, 60, 90, and 120 minutes compared to non-transgenic mice (Figure 3L).

### Protein levels of Tbc1d1 are increased in A315T line 23 and line 61 transgenic mice

**Figure 4:**
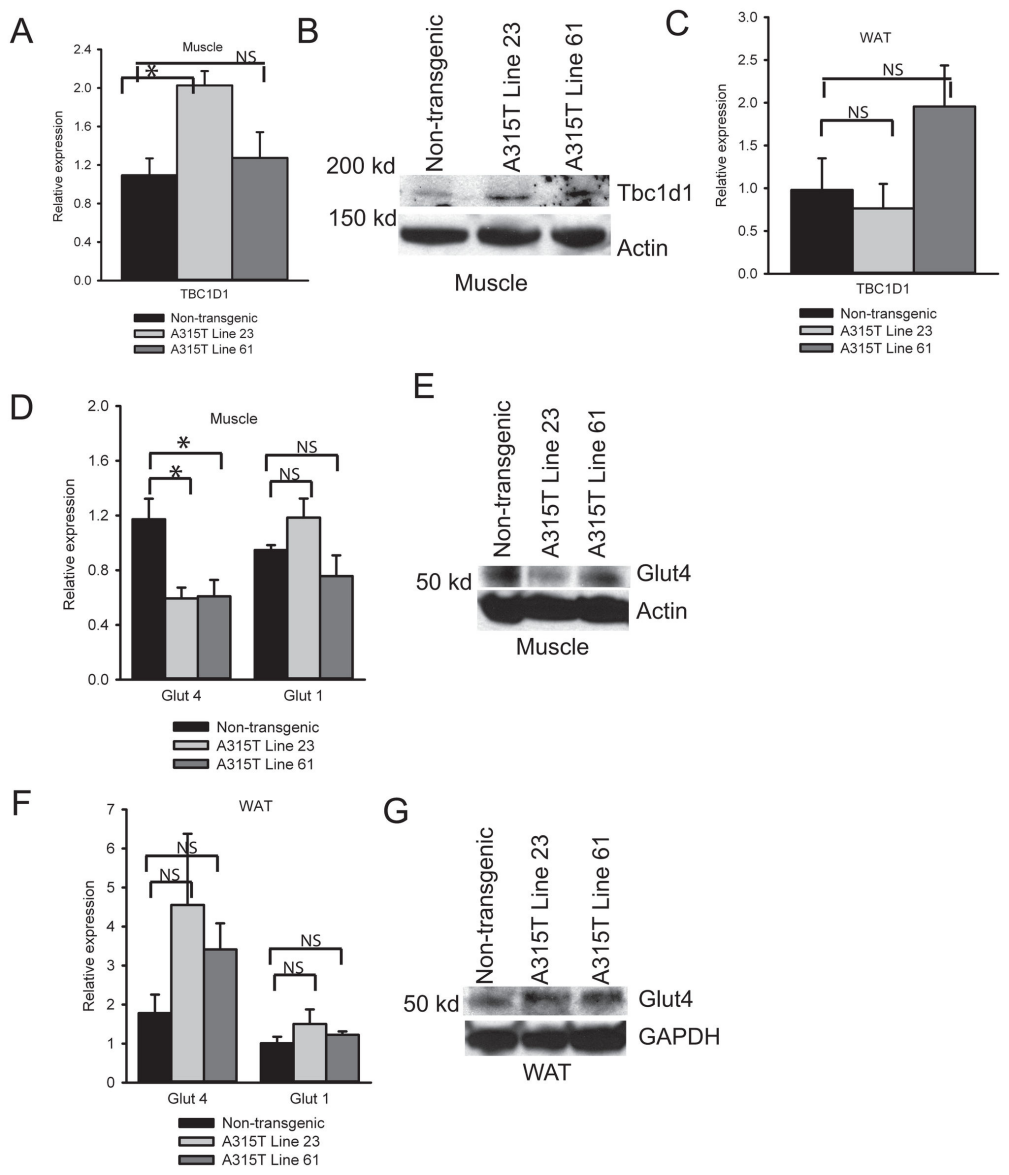
Tbc1d1 and Glut4 levels in skeletal muscle and white adipose tissue. (**A**) Quantification of TBC1D1 mRNA levels in muscle (6 weeks, N=4). Data are fold gene expression normalized with cyclophilin and expressed as mean ± S.E.M. (**B**) Western blot showing protein level of Tbc1d1 in non-transgenic and A315T line 23 and line 61 mice. 50 µg of total protein loaded per lane. (**C**) Quantification of TBC1D11 mRNA levels in white adipose tissue. Data are fold gene expression normalized with cyclophilin and expressed as mean ± S.E.M. (**D**) Quantification of Glut1 and Glut4 mRNA levels in muscle (6 weeks, N=4). Data are fold gene expression normalized with cyclophilin and expressed as mean ± S.E.M. (**E**) Western blot showing protein level of Glut4 in skeletal muscle of non-transgenic, A315T line 23, and A315T line 61 mice. 50 µg of total protein loaded. Actin was used as a loading control. (**F**) Quantification of Glut1 and Glut4 mRNA levels in white adipose tissue (6 weeks, N=4). Data are fold gene expression normalized with cyclophilin and expressed as mean±S.E.M. (**G**) Western blot showing protein level of Glut4 in WAT of non-transgenic, A315T line 23, and A315T line 61. 50 µg of total protein loaded. GAPDH was used as a loading control. Representative western blots are shown, each sample was run ≥ 2 times with N ≥ 2 mice in each group analyzed. Statistical significance between non-transgenic and A315T mice: *P<0.05.

We then sought to better understand mechanisms underlying the obese phenotype of line 61 TDP-43 transgenic mice. If TDP-43 knockouts showed decreases in skeletal muscle levels of Tbc1d1, a key regulator of glucose homeostasis and obesity, we hypothesized that TDP-43 over-expression might result in elevated steady state levels of Tbc1d1. Tbc1d1 mRNA levels were significantly increased in line 23 compared to non-transgenic mice (Figure 4A). On western blotting, Tbc1d1 steady state protein levels were increased in the skeletal muscle in both line 23 and line 61 mice compared to controls (Figure 4B). In adipose tissues there was no significant change in Tbc1d1 mRNA expression levels in either transgenic line compared to non-transgenic mice (Figure 4C). These results indicate that TDP-43 over-expression results in increased steady state levels of Tbc1d1 protein in skeletal muscle and in conjunction with conditional knockout experiments show that TDP-43 is a regulator of Tbc1d1 muscle levels in vivo.

### Over-expression of TDP-43 in muscle leads to Glut4 translocation abnormalities in skeletal muscle

Because Tbc1d1 is known to be involved in pathways regulating insulin mediated glucose uptake in skeletal muscle via effects on Glut4 translocation, we asked whether TDP-43 transgenic mice manifested physiologic alterations in glucose homeostasis. We first assessed whether line 23 and line 61 mice showed changes in Glut4 levels. qPCR analysis showed a small but significant decrease in mRNA levels for Glut4 in transgenicskeletal muscle compared to controls with no change in the mRNA level of Glut1 (Figure 4D). However, Glut4 steady state protein levels were decreased in line 23 and unchanged between line 61 mice and controls (Figure 4E). In white adipose tissues, mRNA and protein levels of Glut4 and Glut1 were unchanged between the transgenic lines and non-transgenic mice (Figure 4 F, G).

**Figure 5:**
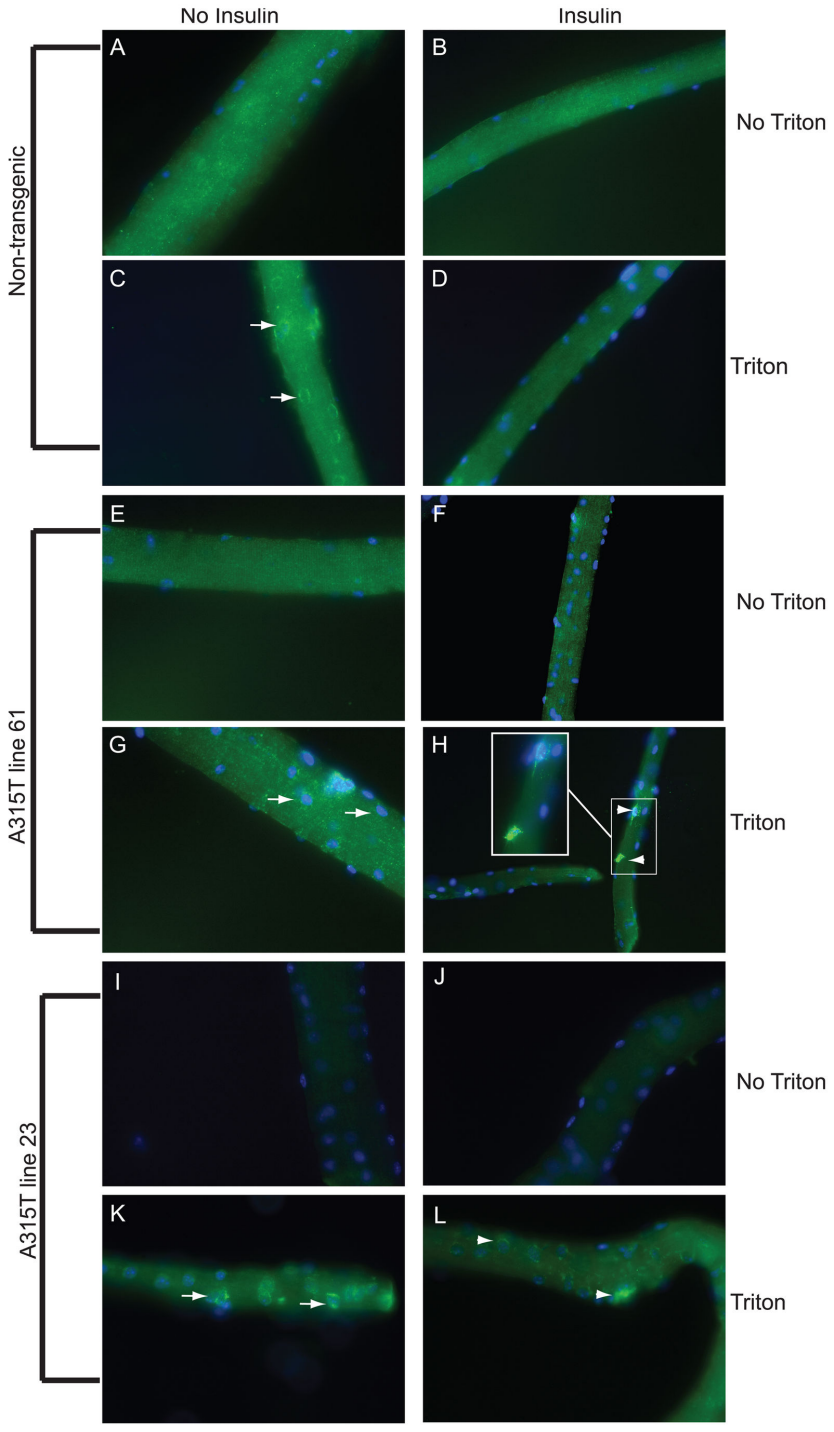
Glut4 translocation assay in isolated flexor digitorum brevis muscle. (**A**–**L**) Glut4 immuno-reactivity in FDB fibers from non-transgenic mice (**A**–**D**), A315T line 61 (E–H), and A315T line 23 (I–L). Arrows highlight peri-nuclear Glut4 containing vesicles without insulin treatment. Arrowheads indicate dense accumulation of Glut4 immuno-reactivity with insulin treatment. Mice were 8-12 weeks of age. Magnification =400x, except for (**H**) which is 200X (insert =400X).

To determine whether line 61 mice showed deficits in insulin mediated Glut4 translocation from the peri-nuclear region to cell surface, we employed a Glut4 translocation assay performed in isolated flexor digitorum brevis (FDB) muscle [23]. In this assay, intact FDB muscle fibers isolated from 8–12 week old mice are subject to insulin treatment, followed by fixation with or without permeabilizing agents, and then immuno-stained with an amino terminal directed Glut4 antibody. Permeabilization allows entry of antibody into the muscle to access peri-nuclear localizing pools of Glut4 containing vesicles. Glut4 containing peri-nuclear vesicles are not visualized without permeabilization. In non-transgenic mice, in the absence or presence of insulin, non-permeabilized muscle fibers show diffuse punctate Glut4 immuno-staining, likely reflecting Glut4 localization on the cell surface (Figure 5, A and B and Figure S4A). Permeabilized muscle fibers show clear foci of peri-nuclear punctate immuno-reactivity, corresponding to well described Glut4 containing vesicles, which are depleted following insulin treatment (Figure 5, C and D and Figure S4, B and C). Like non-transgenic controls, line 61 non-permeabilized muscle fibers show diffuse punctate Glut4 immuno-reactivity with or without insulin (Figure 5, E and F). However, in stark contrast to controls, muscle fibers from line 61 mice treated with insulin and then permeabilized show retention and even dense accumulation of peri-nuclear Glut4 immuno-reactivity (Figure 5, G and H and Figure S4D). Muscle fibers from A315T line 23 showed a similar pattern of impaired Glut4 translocation in response to insulin treatment (Figure 5 I–L). These results indicate that over-expression of A315T TDP-43 in skeletal muscle leads to aberrant translocation of peri-nuclear Glut4 containing vesicles in response to insulin.

We also wished to determine whether over-expression of wild type human TDP-43 in skeletal muscle in vivo would yield similar Glut4 translocation abnormalities. For this analysis, we selected wild type TDP-43 transgenic line 4. Although these mice develop an early onset severe myopathy [13], we believed that because this line expresses high levels of TDP-43 in skeletal muscle, it would manifest similar abnormalities in Glut4 translocation in response to insulin. The Glut4 translocation assay performed in skeletal muscle from WT TDP-43 transgenic line 4 was abnormal, showing both preservation of peri-nuclear Glut4 containing vesicles, as well as a marked accumulation of internalized Glut4 immuno-reactivity (Figure S5, A-C and Figure S4E). These findings demonstrate that both wild type and A315T TDP-43 over-expression in skeletal muscle results in abnormal Glut4 translocation in response to insulin.

### Glut4 translocation in adipocytes remains insulin responsive

**Figure 6:**
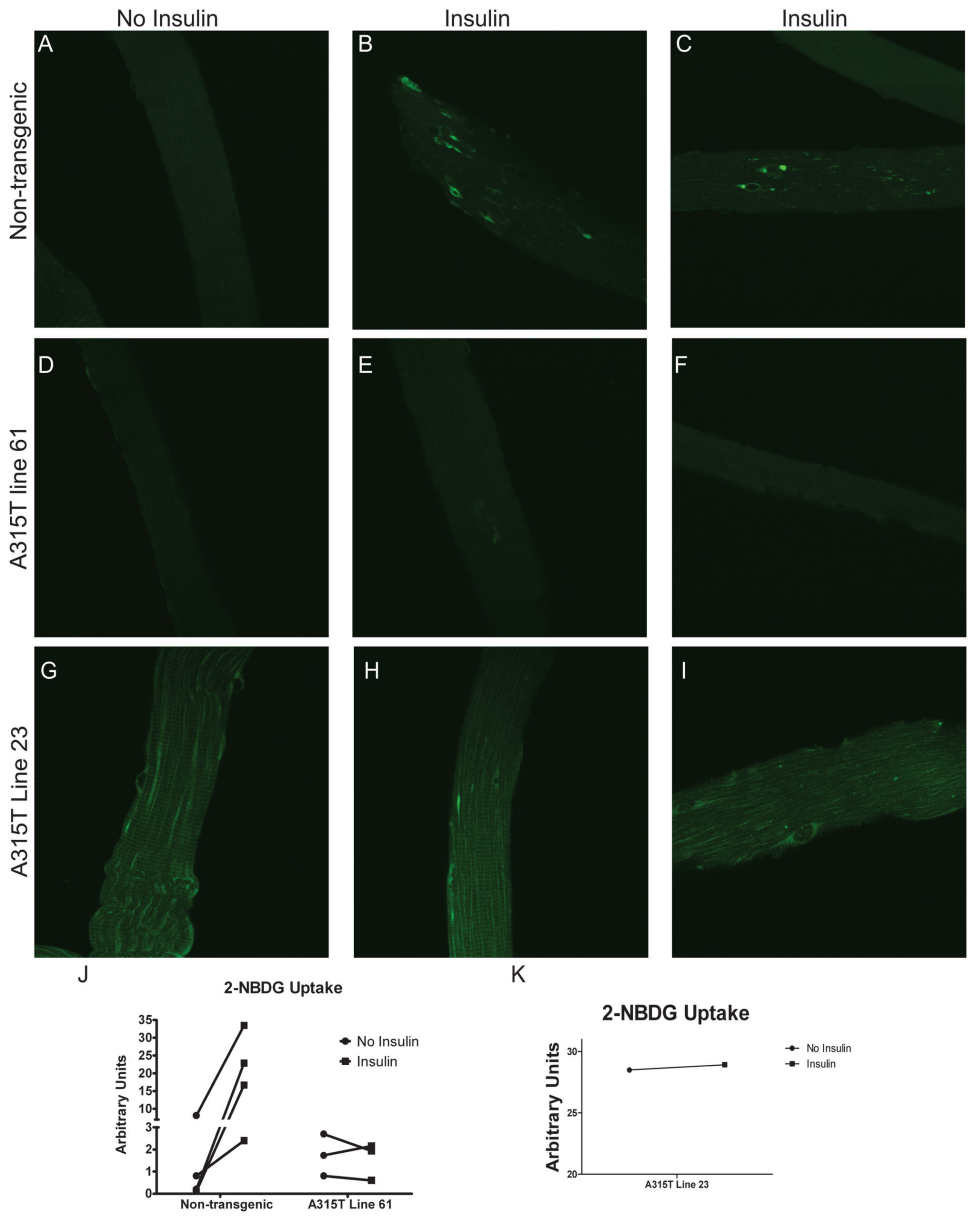
Glucose uptake assay in live flexor digitorum brevis muscle fibers. (**A**–**I**) Confocal images of 2-NBDG exposed live FDB fibers from non-transgenic (**A**–**C**), A315T line 61 (D–F), and A315T line 23 (G–I) mice with and without insulin. (**J**) Change in fluorescence (arbitrary units) with the addition of insulin to 2-NBDG exposed FDB fibers from non-transgenic and A315T line 61 mice. (K) Change in fluorescence (arbitrary units) with the addition of insulin to 2-NBDG exposed FDB fibers to A315T line 23 mice, Mice were 8-12 weeks of age. Each line represents a paired individual experiment with 4-15 fibers measured for each experimental condition. Magnification = 400X.

To address whether TDP-43 effects insulin mediated Glut4 translocation in other tissues, we isolated adipocytes from the gonadal fat pad of 6-8 week old transgenic and non-transgenic mice and performed a Glut4 translocation assay. Treatment with insulin resulted in comparable increases in Glut4 immuno-reactivity at the cell surface of adipocytes from both non-transgenic and transgenic mice (FigureS 6). Of note, the adipocytes in A315T line 61 were larger than in the non-transgenic mice. This increase in adipocyte size may account for the increase of basal levels of Glut4 immunoreactivity. To insure that Glut4 only at the cell surface was visualized by antibody, we did not permeabilize the adipocytes with Triton-X for immunofluorescence. Thus, in contrast to skeletal muscle, adipocytes from TDP-43 line 61 transgenic mice retain their Glut4 translocation responsiveness to insulin.

### A315T transgenic mice have abnormal glucose uptake in skeletal muscle

To understand whether the observed changes in Glut4 translocation affected glucose uptake into skeletal muscle in response to insulin, we performed a glucose uptake assay in FDB muscle fibers utilizing a fluorescently tagged glucose analog [24]. In response to insulin administration, isolated FDB muscles from non-transgenic controls incubated with 2-NBDG showed an increase in fluorescence compared to non-insulin treated fibers (Figure 6, A-C and J). In contrast, isolated FDB fibers from line 61 TDP-43 transgenic mice incubated with 2-NBDG failed to show a significant increase in fluorescence following insulin administration (Figure 6, D–F and J). Muscle fibers from A315T line 23 show similar results of abnormal 2-NBDG uptake in response to insulin administration (Figure 6, G-I and K). These results indicate that TDP-43 over-expression in skeletal muscle results in marked impairment of glucose uptake in response to insulin, confirming the physiological impact of altered Glut4 translocation. Thus, TDP-43 has a critical role in skeletal muscle glucose homeostasis in vivo.

To ensure that abnormal glucose uptake is specific for TDP-43 over-expression and not related to expression of a toxic ALS related protein in general, we performed the 2-NBDG assay in FDB muscle fibers isolated from transgenic mice expressing G93A Cu, Zn superoxide dismutase (SOD1) which causes familial ALS type 1 [25]. In contrast to the results observed in A315T transgenic mice, 2-NBDG fluorescence increased within live FDB muscle fibers from G93A SOD1 mice following insulin treatment similar to what was observed in non-transgenic controls (Figure S7). Thus, glucose uptake abnormalities in skeletal muscle appear to be specific for TDP-43 over-expression and not just related to expression of a motor neuron disease related protein.

## Discussion

The results from this study indicate that over-expression of TDP-43 can lead to significant weight gain, increased fat deposition, adipocyte hypertrophy, and abnormal insulin mediated Glut4 translocation in skeletal muscle. These metabolic phenotypes do not appear related to CNS expression of TDP-43 but rather result from TDP-43 expression in peripheral target organs. Skeletal muscle represents one such target where TDP-43 acts as a key regulator of glucose uptake in response to insulin, potentially via effects on Tbc1d1. The loss of TDP-43 from skeletal muscle results in diminished Tbc1d1 mRNA and protein levels while we show here that TDP-43 over-expression results in elevated Tbc1d1 levels [19]. In human beings, Tbc1d1 has been linked to familial obesity syndromes while in mice the loss of Tbc1d1 produces leanness and conveys resistance to diet induced obesity [21,26,27]. Whether TDP-43 acts in skeletal muscle alone or in other potential target organs, such as fat, to produce the complete obesity phenotype is not clear and will likely require target specific expression or knockdown to resolve.

Both fat and skeletal muscle normally will respond to insulin with increased glucose uptake via the translocation of Glut4 onto the cell surface. However, our results here show that TDP-43 over-expression inhibits the insulin mediated mobilization of Glut4 in skeletal muscle but not in white adipocytes suggesting differential regulation of Glut4 trafficking. Although both cell types utilize PI-3 and Akt signaling cascades for effect, the role of certain substrates has been shown to be cell type specific. *In vivo* alterations of the Rab GTPase activating protein AS160 (Alt substrate of 160kDA, or TBC1D4) is associated with reduced Glut4 translocation and glucose transport in skeletal muscle in response to insulin but not in adipocytes [28–30].

TDP-43 has been linked to both familial and sporadic cases of ALS. Clinicians have noted that ALS patients may lose significant weight and muscle mass despite having adequate caloric intake and have correlated this cachexia with poor survival [31]. The mechanisms underlying ALS cachexia are unknown, although there is extensive literature documenting metabolic derangements in patients with sporadic ALS [32]. ALS patients demonstrate hyper-metabolism with a marked increase in measured resting energy expenditure, a loss of muscle mass, and a gain of fat mass despite being in caloric balance [33–35]. Such data argue strongly for a primary metabolic abnormality in sporadic ALS that can be readily distinguished from poor caloric intake. In addition, there is evidence of glucose homeostasis aberrations in sporadic ALS who show impaired glucose tolerance and insulin resistance [36,37]. The molecular basis for energy derangement and fat/lean mass changes in sporadic ALS are unknown. However, the recent recognition of a key role for TDP-43 in sporadic ALS, and its novel function as a regulator of body mass composition and glucose homeostasis pose the question as to whether such changes observed in sporadic ALS patients are a direct consequence of TDP-43 actions on muscle/fat metabolic parameters.

## Methods

### Ethics Statement

All mouse experiments were conducted in accordance with the National Institute of Health Guide for the Care and Use of Laboratory Animals. The University of Texas Southwestern Medical Center Institutional Animal Care and Use Committee (IACUC) approved all animal procedures with protocol 2008-0207.

### Mice

TDP-43 A315T line 61 transgenic mice were generated as previously described. The transgene was injected into C57Bl6/SJL F1 hybrids and founders were bred to CD1 mice (Charles Rivers, Wilmington, MA) [13]. . Mice were maintained on a mixed background by breeding to non-transgenic littermates or CD1 mice The lethal motor phenotypes in A315T TDP-43 line 23 and WT TDP-43 line 4 mice have also been well characterized. Briefly, the prion promoter was used to drive expression of human TDP-43 cDNA encoding either the wild-type or familial ALS linked A315T mutant. Mice were genotyped for the presence of the transgene by PCR using the following primers: Forward 5’ GGTGGTGGGATGACCTTTGG3’ and Reverse 5’ GTGGATAACCCCTCCCCCAGCCTAGAC3’. G93A SOD1 transgenic mice have been previously well characterized [25]. Mice were fed standard chow *ad libitum* (Teklad Global 16% Protein Diet #2916). All mice used in this study were male.

### Body weight and body composition

Mice were weighed and body fat composition was determined using an EchoMRI-100™ (Echo Medical Systems, Houston, Texas). Body fat and lean mass percentage are calculated based on total body mass.

### Glucose tolerance test

Mice were fasted overnight (16 hours), weighed and injected ip with glucose at 1mg/g body weight. Glucose measurements were taken at 0, 15, 30, 60, 90, and 120 minutes using a Precision Xtra Glucose Meter (Abbott Diabetes Care, Inc., Alameda, CA).

### Blood collection

Mice were fasted overnight (16 hours) and facial vein blood was collected using Goldenrod animal lancet (Medipoint, Mineola, New York). Serum was obtained by allowing the blood to clot at room temperature for 30 minutes and then centrifuging at 4000 rpm for 10 minutes. Fasting glucose, cholesterol, triglycerides, GGT, and CK measurements were analyzed using a Vitros 250 (Ortho Clinical Diagnostics, Raritan, NJ) in the UT Southwestern Metabolic Phenotyping Core.

### Metabolic chambers

Mice were transferred to the UT Southwestern Metabolic Phenotyping Core and allowed to acclimate for 5 days in the metabolic chambers (TSE Systems, Inc., Chesterfield, MO). Data was then collected for 96 hours. Mice were 10 weeks of age at the time of data collection. 6 transgenic and 6 non-transgenic age matched littermates were studied.

### Real time quantitative PCR

Quantitative PCR was performed as previously described [38]. Primer sequences are available from the authors upon request.

### Statistical analysis

Data was presented as means ±S.E.M. We used a two-tailed t test to determine P values for statistical significance (Microsoft Excel and GraphPad Prism). Analysis of covariance (ANCOVA) using energy expenditure (EE) as the dependent variable, genotype as the fixed factor, and body composition, activity, and intake as covariates was calculated using IBM SPSS V.19.0.0 as previously described [39].

### Western blots

Tissues were homogenized in 1 ml of 1X subcell buffer (G‐Biosciences) with 10µl of Sigma Protease Inhibitor Cocktail as previously described [13]. For loading controls, actin (muscle), beta III tubulin (spinal cord), and GAPDH (fat) were used.

### Immunofluorescence

Immunofluorescence was performed as previously described [40].

### Antibodies

Antibodies are from the following companies: TDP-43 polyclonal (10782-2AP, recognizes human and mouse TDP-43) and monoclonal (60019-2-Ig, recognizes human TDP-43) (Protein Tech Group), Tbc1d1 (CAT#5929S, recognizes G689, Cell Signaling), Glut4 N-terminal (SC-1606, recognizes N-20) and GAPDH(SC-25778) (Santa Cruz), monoclonal ubiquitin (MAB1510, Millipore), actin (CAT#A2066, Sigma), beta III tubulin (CAT#2020-TUB, PhosphoSolutions), and polyclonal ubiquitin (CAT#Z0458) and GFAP (CAT#Z0334) (Dako).

### Muscle histology

Muscle H&E was performed using standard protocols as previously described [13].

### Muscle fiber preparation

Single flexor digitorum brevis (FDB) muscle fibers were isolated by enzymatic digestion and mechanical disruption of intrinsic foot muscles from 8 to 10 week old mice [41]. The muscles were rapidly dissected and placed in DMEM plus collagenase type 2 (Worthington, 100mg/ml) and incubated at 37^°^ C for 90 minutes. Fibers were dissociated by triturating in a wide-bore pipette, pelleted by centrifugation and re-suspended in DMEM plus 10% fetal bovine serum, 1% glutamine, 100U/ml penicillin-streptomycin and 10mM HEPES. Approximately 100 to 200 fibers were plated in 35 mm plates and the cultures maintained for 20 hours.

### Glut4 transporter translocation assay in muscle

After an overnight culture, FDB muscle fibers were switched to serum free media for three hours. 300 nM of insulin was then added to some fibers for 30 minutes. Then all fibers were washed with PBS and fixed with 2% buffered paraformaldehyde, with or without permeabilization in 0.25% triton X-100 for 5 min at room temperature and incubated with anti-GLUT4 antibody. The slides were then incubated with the appropriate secondary antibody. Slides were viewed used with an E800 fluorescent microscope (Nikon). Confocal images were acquired on a Leica SP5 laser scanning confocal microscope in the UT Southwestern Microscopy Core.

### Glut4 transporter translocation assay in isolated adipocytes

Gonadal fat pads isolated from 6–8 week old male TDP-43 transgenic and non-transgenic mice were digested in collagenase type I (1.5mg/ml) followed by gentle shaking at 37 degrees for 1.5 hours, filtration through a 210 micron filter, and then centrifuged at 1200 rpm for 5 minutes. Mature adipocytes were by then pulled off top. Isolated adipose cells were equally distributed in plastic vials with in DMEM/Ham’s F-12 medium and incubated without or with 600nM insulin at 37C for 30 minutes following published methods [42]. Adipocytes were washed with PBS and fixed with 4% buffered paraformaldehyde at room temperature and incubated with anti-GLUT4 antibody but without Triton-x. The cells were then incubated with the appropriate secondary antibody. Confocal images were acquired on a Leica SP5 laser scanning confocal microscope in the UT Southwestern Microscopy Core. Quantification of fluorescence was done using ImageJ software.

### Glucose uptake in single FDB fibers

Glucose uptake was performed as previously described with modifications [24]. After overnight culture, FDB muscle fibers were switched to serum free media for three hours before incubation with a glucose-free (2mmol/l pyruvate) medium containing 50µmol/2-NBDG for 15min and then washed for 20 min. When used, 300 nM of insulin was present during the last 15 minutes of the first washing period and during the second exposure to 2-NBDG. Multiple fibers were imaged for each condition using a Zeiss LSM150 confocal microscope (UT Southwestern Microscopy Core) with excitation at 488 nm and emission at 515nm. Quantification of the images was done using ImageJ software.

## Supporting Information

Figure S1 **Quantification of adipose dysfunction markers in WAT by qPCR.** (8-12 weeks, N=5). Data are fold gene expression normalized with cyclophilin and expressed as mean ± S.E.M.
(TIF)

Figure S2 **A315T line 61 mice show no spinal cord pathology at 16 weeks of age.** TDP-43 and ubiquitin immuno-reactivity in lumbar spinal cord sections from adult non-transgenic (**A**,**C**) and line 61 (B,D) mice. A monoclonal TDP-43 antibody recognizing human TDP-43 is used in A and B (green). A polyclonal TDP-43 antibody recognizing human and mouse TDP-43 is used in **C** and **D** (red). (**E**) A315T line 61 spinal cord sections immuno-stained with monoclonal TDP-43 (green) and GFAP (red) antibodies. Magnification = 200X.
(TIF)

Figure S3 **Metabolic cage data for individual mice.** (**A**) Energy expenditure normalized to lean mass. (**B**) Weight of mice used in metabolic cages. (**C**) Lean mass of each mouse. (**D**) Fat mass of each mouse. For each graph, each value is an individual mouse. N=6 in each group. Mice were 10 weeks of age. Bar=mean measurements ±S.E.M.
(TIF)

Figure S4 **(A–B) Confocal images showing Glut4 immuno-reactivity in isolated FDB fibers from non-transgenic mice without (A) or with (B) permeabilization.** Note the visualization of Glut4 punctate immuno-reactivity in peri-nuclear foci following permeabilization. (**C**–**E**) Confocal images showing Glut4 immuno-reactivity in isolated FDB fibers from non-transgenic (**C**), A315T line 61 (D) and TDP-43 WT line 4 (E) following insulin treatment and permeabilization. Mice were 8-12 weeks of age. Magnification = 630X.
(TIF)

Figure S5 **Glut4 translocation assay in isolated flexor digitorum brevis muscle from TDP-43 WT line 4 mice.** (**A**–**C**) Glut4 immuno-reactivity in FDB fibers from TDP-43 WT line 4 Mice were 12 weeks of age. Arrows highlight peri-nuclear Glut4 containing vesicles without insulin treatment. Arrowheads indicate dense accumulation of Glut4 immuno-reactivity with insulin treatment. Magnification =400X.
(TIF)

Figure S6 **Glut4 localization on the cell surface of isolated adipocytes.** (**A**–**B**) Glut4 immuno-reactivity on the cell surface of isolated adipocytes from non-transgenic mice without insulin (**A**) and treated with insulin (**B**). (**C**–**D**) Glut4 immuno-reactivity on the cell surface of isolated adipocytes from A315T line 61 mice without insulin (**C**) and treated with insulin (**D**). Non-permeabilized adipocytes were used, so only Glut4 at the cell surface was visualized by the antibody using confocal microscopy. (**E**) Quantification of Glut4 immunofluorescence in adipocytes. N=10-12 cells per group. Error bars are ±S.E.M. *** > .0001. Note the larger adipocytes from A315T line 61 transgenic mice (**C**–**D**). Mice were 12 weeks of age. Magnification = 630X.
(TIF)

Figure S7 **Glucose uptake assay in live flexor digitorum brevis muscle fibers from G93A SOD1 mice.** (**A**–**C**) Confocal images in 2-NBDG exposed live FDB fibers from 8 week old G93A SOD1mice with and without insulin. (**D**) Change in fluorescence (arbitrary units) with the addition of insulin to 2-NBDG exposed FDB fibers from G93A SOD1 mice. Line represents a paired experiment with 10 fibers measured for each experimental condition. Mice were 12 weeks old. Magnification = 400X.
(TIF)

Table S1 **ANCOVA of data from metabolic cages.** Energy expenditure (24 hour) was set as the dependent variable, with lean mass, total activity, and food as the covariates.
(TIF)
