# Supplementary figures and images for: TDP-43, an ALS Linked Protein, Regulates Fat Deposition and Glucose Homeostasis
Source: PLoS One. 2013 Aug 13;8(8):e71793. doi: 10.1371/journal.pone.0071793 (PMC3742534; doi:10.1371/journal.pone.0071793)

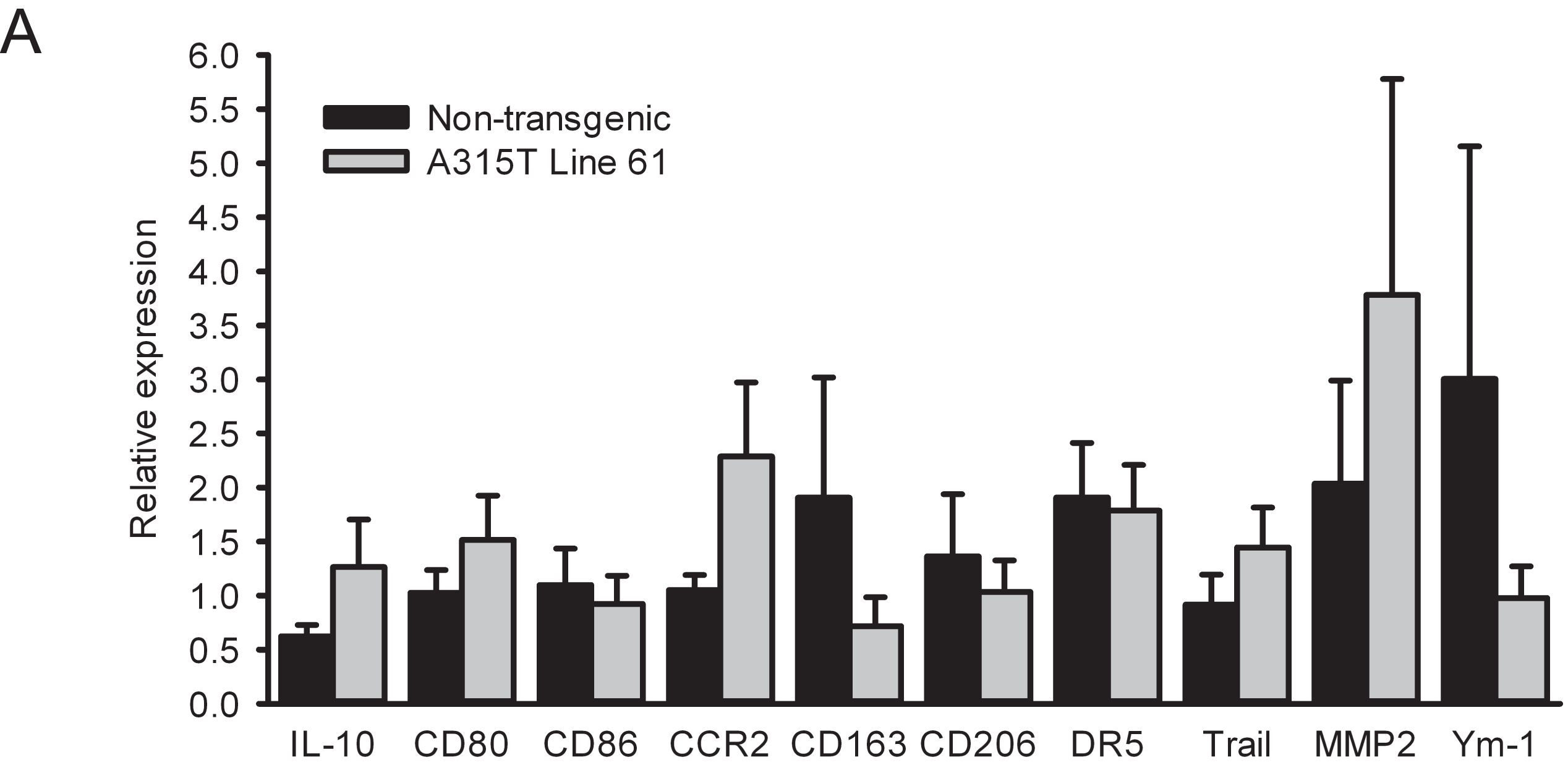

Supplement: Figure S1 — Quantification of adipose dysfunction markers in WAT by qPCR. (8-12 weeks, N=5). Data are fold gene expression normalized with cyclophilin and expressed as mean ± S.E.M. (TIF) [file pone.0071793.s001.tif]

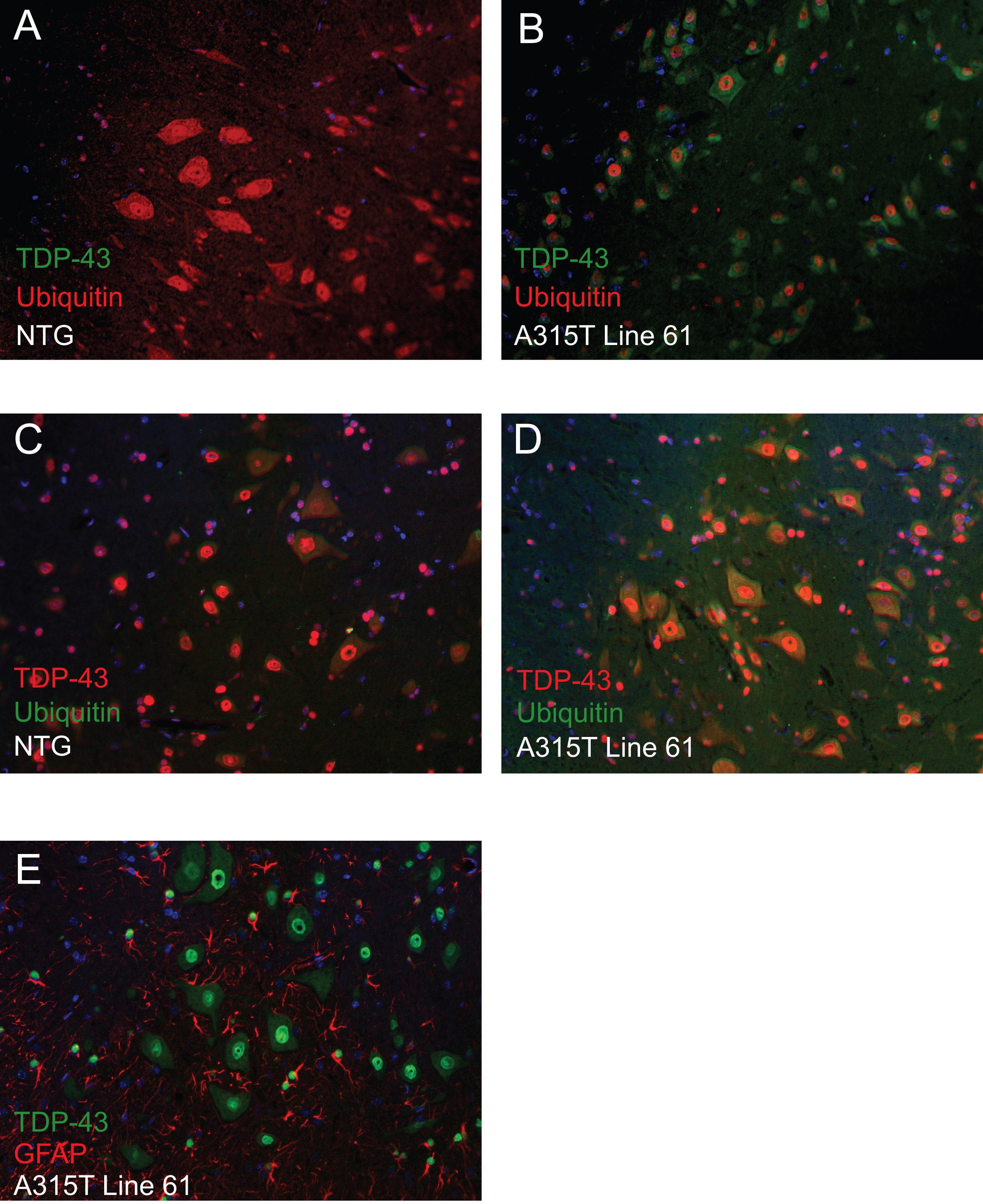

Supplement: Figure S2 — A315T line 61 mice show no spinal cord pathology at 16 weeks of age. TDP-43 and ubiquitin immuno-reactivity in lumbar spinal cord sections from adult non-transgenic (A,C) and line 61 (B,D) mice. A monoclonal TDP-43 antibody recognizing human TDP-43 is used in A and B (green). A polyclonal TDP-43 antibody recognizing human and mouse TDP-43 is used in C and D (red). (E) A315T line 61 spinal cord sections immuno-stained with monoclonal TDP-43 (green) and GFAP (red) antibodies. Magnification = 200X. (TIF) [file pone.0071793.s002.tif]

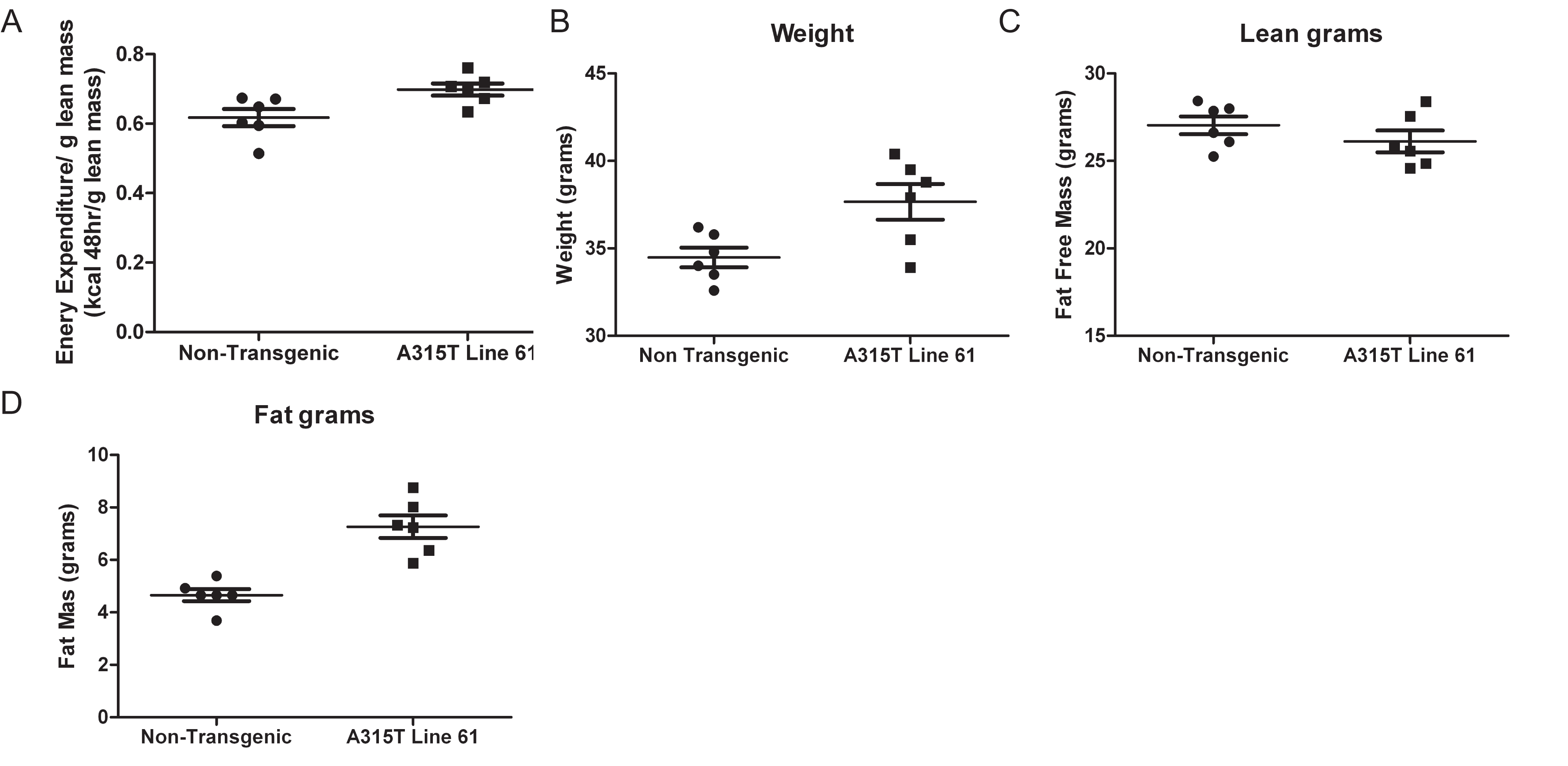

Supplement: Figure S3 — Metabolic cage data for individual mice. (A) Energy expenditure normalized to lean mass. (B) Weight of mice used in metabolic cages. (C) Lean mass of each mouse. (D) Fat mass of each mouse. For each graph, each value is an individual mouse. N=6 in each group. Mice were 10 weeks of age. Bar=mean measurements ±S.E.M. (TIF) [file pone.0071793.s003.tif]

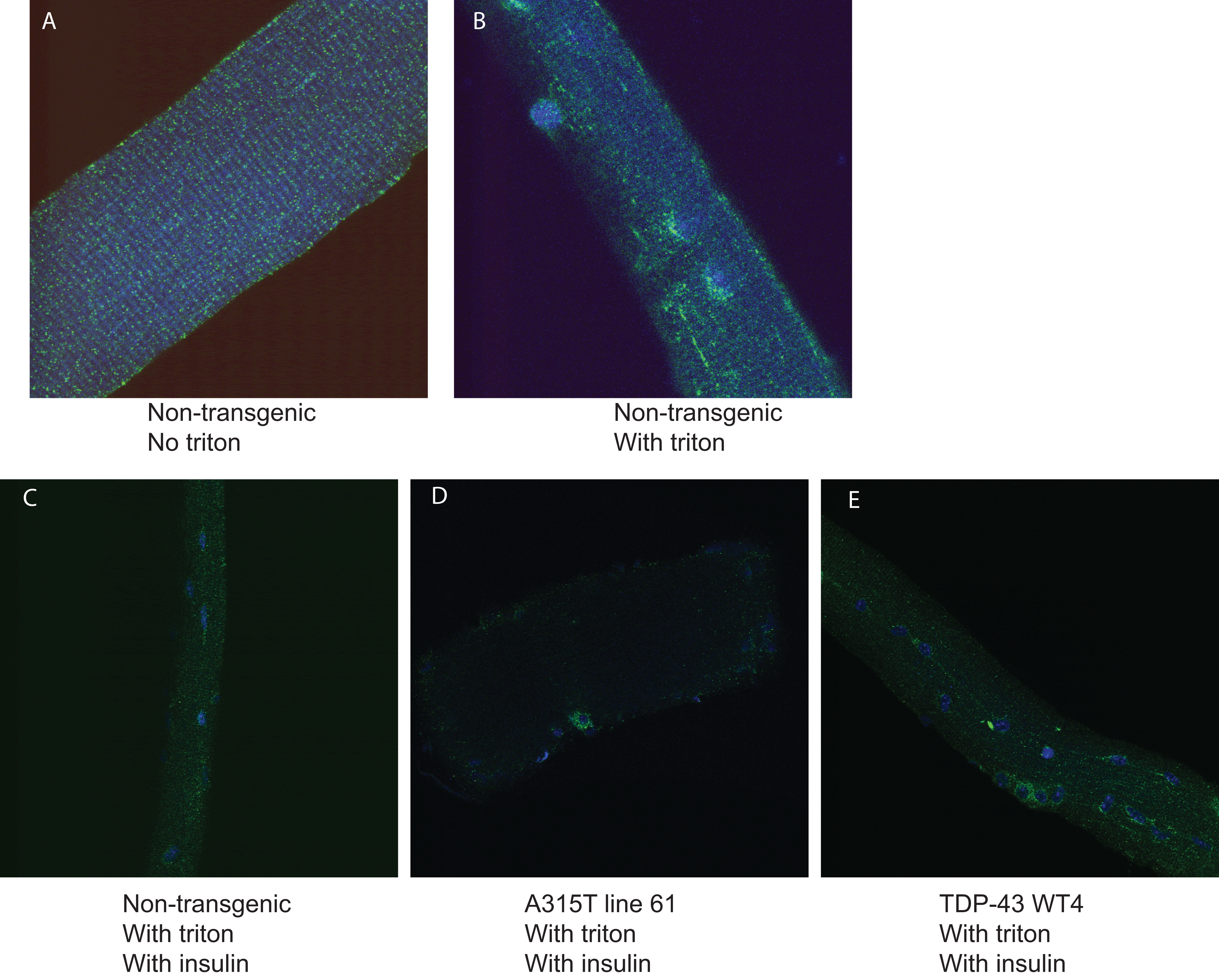

Supplement: Figure S4 — (A–B) Confocal images showing Glut4 immuno-reactivity in isolated FDB fibers from non-transgenic mice without (A) or with (B) permeabilization. Note the visualization of Glut4 punctate immuno-reactivity in peri-nuclear foci following permeabilization. (C–E) Confocal images showing Glut4 immuno-reactivity in isolated FDB fibers from non-transgenic (C), A315T line 61 (D) and TDP-43 WT line 4 (E) following insulin treatment and permeabilization. Mice were 8-12 weeks of age. Magnification = 630X. (TIF) [file pone.0071793.s004.tif]

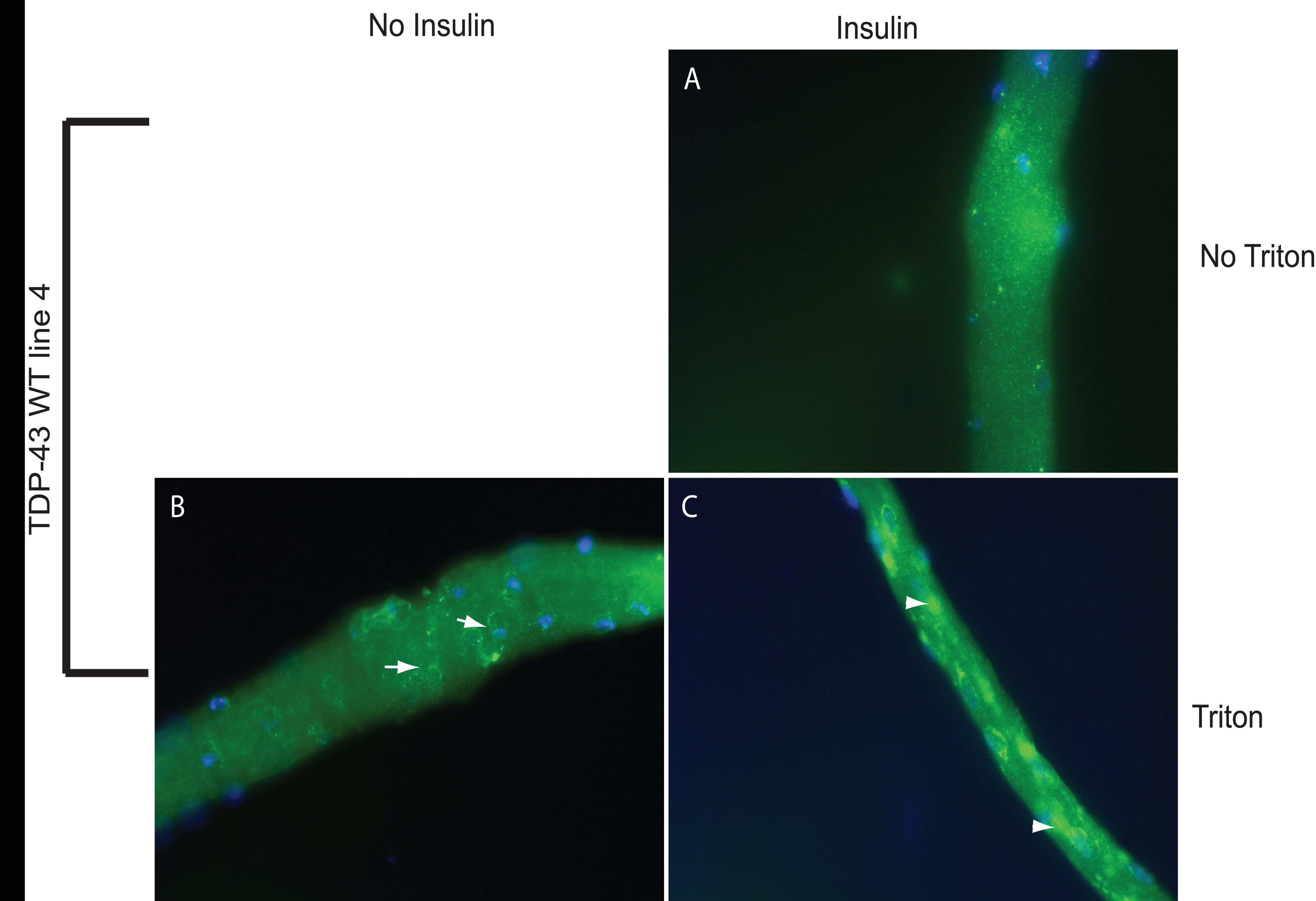

Supplement: Figure S5 — Glut4 translocation assay in isolated flexor digitorum brevis muscle from TDP-43 WT line 4 mice. (A–C) Glut4 immuno-reactivity in FDB fibers from TDP-43 WT line 4 Mice were 12 weeks of age. Arrows highlight peri-nuclear Glut4 containing vesicles without insulin treatment. Arrowheads indicate dense accumulation of Glut4 immuno-reactivity with insulin treatment. Magnification =400X. (TIF) [file pone.0071793.s005.tif]

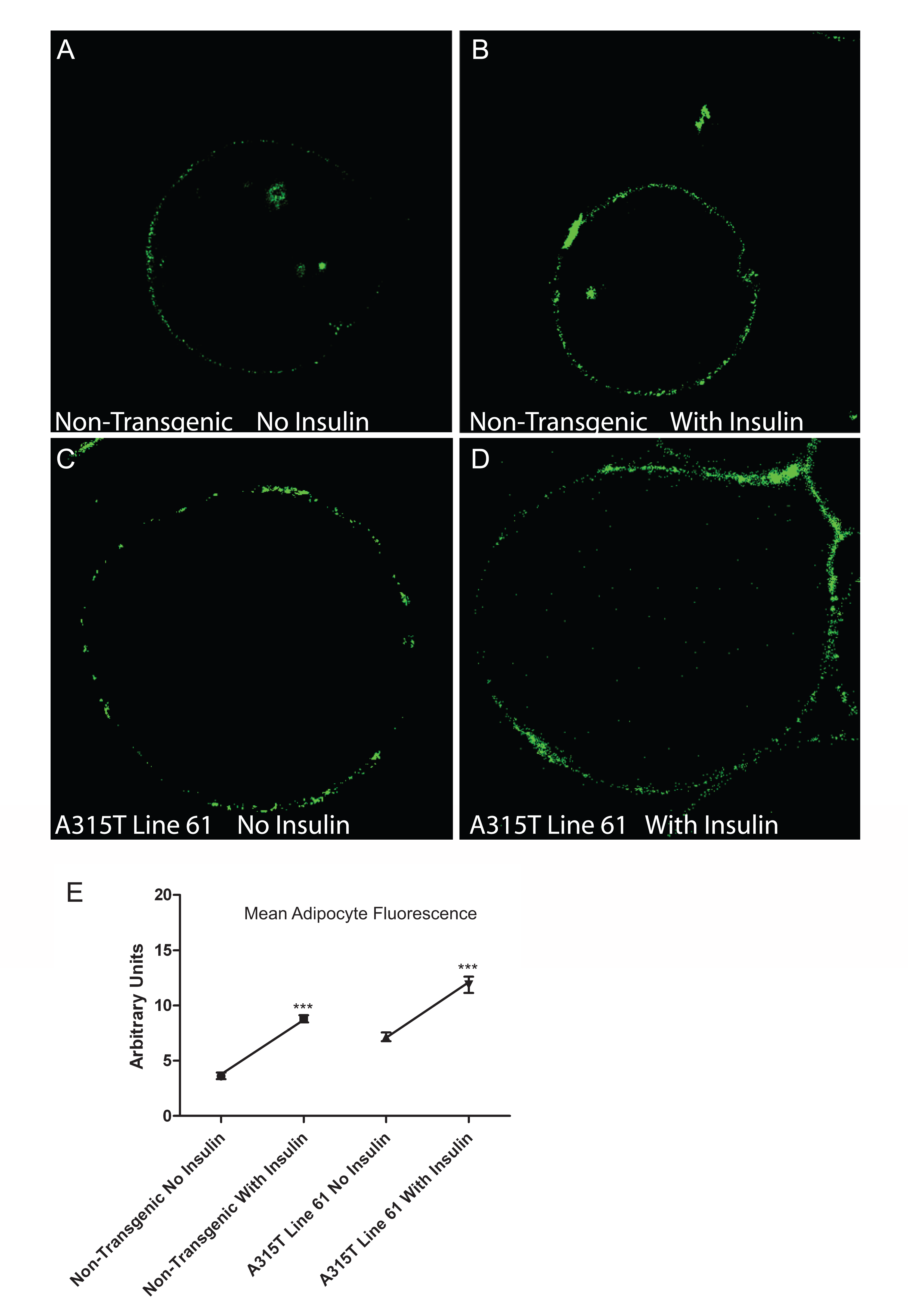

Supplement: Figure S6 — Glut4 localization on the cell surface of isolated adipocytes. (A–B) Glut4 immuno-reactivity on the cell surface of isolated adipocytes from non-transgenic mice without insulin (A) and treated with insulin (B). (C–D) Glut4 immuno-reactivity on the cell surface of isolated adipocytes from A315T line 61 mice without insulin (C) and treated with insulin (D). Non-permeabilized adipocytes were used, so only Glut4 at the cell surface was visualized by the antibody using confocal microscopy. (E) Quantification of Glut4 immunofluorescence in adipocytes. N=10-12 cells per group. Error bars are ±S.E.M. *** > .0001. Note the larger adipocytes from A315T line 61 transgenic mice (C–D). Mice were 12 weeks of age. Magnification = 630X. (TIF) [file pone.0071793.s006.tif]

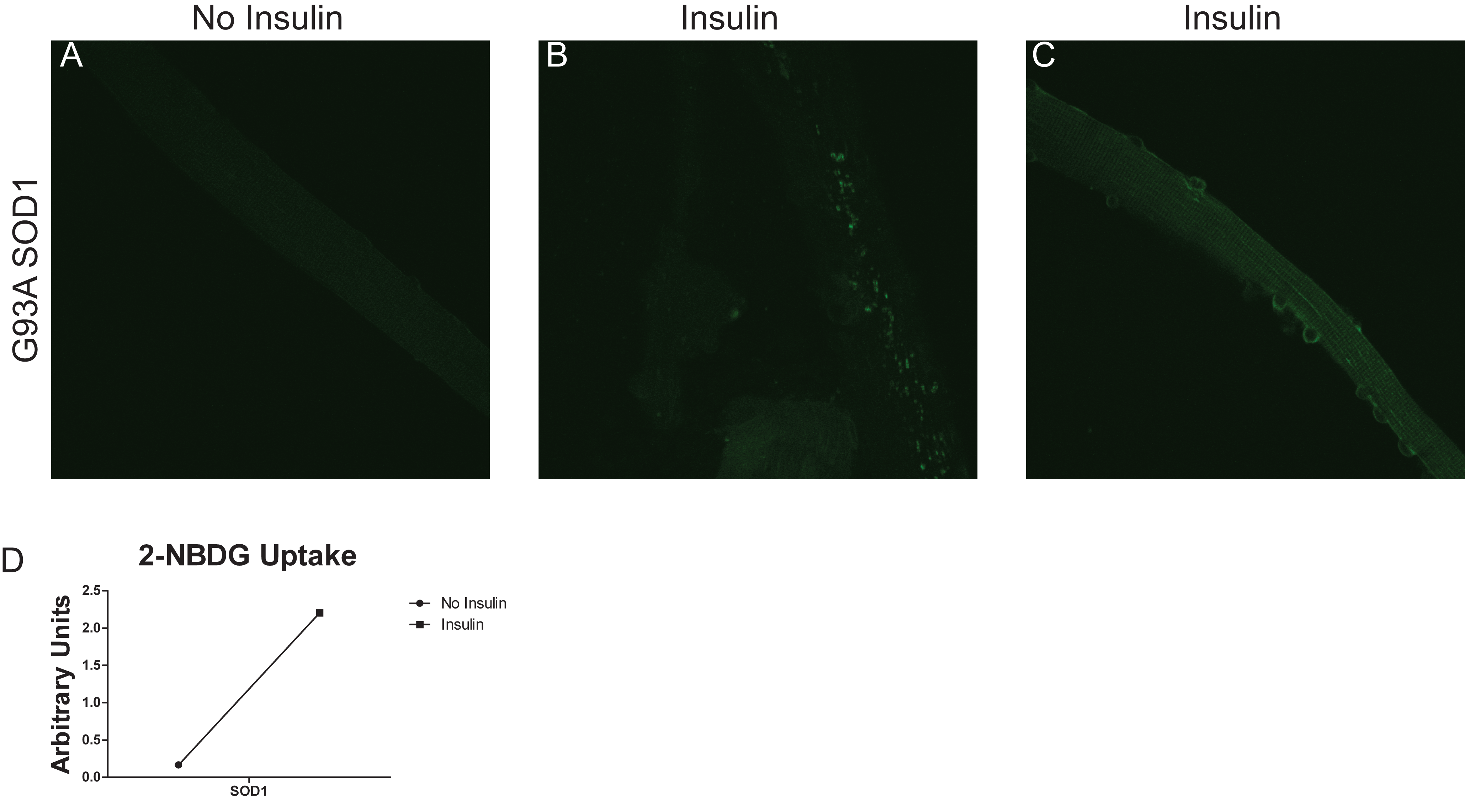

Supplement: Figure S7 — Glucose uptake assay in live flexor digitorum brevis muscle fibers from G93A SOD1 mice. (A–C) Confocal images in 2-NBDG exposed live FDB fibers from 8 week old G93A SOD1mice with and without insulin. (D) Change in fluorescence (arbitrary units) with the addition of insulin to 2-NBDG exposed FDB fibers from G93A SOD1 mice. Line represents a paired experiment with 10 fibers measured for each experimental condition. Mice were 12 weeks old. Magnification = 400X. (TIF) [file pone.0071793.s007.tif]

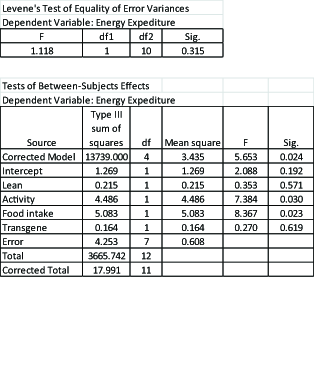

Supplement: Table S1 — ANCOVA of data from metabolic cages. Energy expenditure (24 hour) was set as the dependent variable, with lean mass, total activity, and food as the covariates. (TIF) [file pone.0071793.s008.tif]
